# Progress report on development of classification criteria for adult and juvenile idiopathic inflammatory myopathies

**DOI:** 10.1186/1546-0096-12-S1-P94

**Published:** 2014-09-17

**Authors:** Clarissa Pilkington, Anna Tjärnlund, Matteo Bottai, Victoria Werth, Marianne de Visser, Lars Alfredsson, Anthony Amato, Richard J Barohn, Matthew Liang, Jasvinder Singh, Frederick W Miller, Lisa Rider, Ingrid E Lundberg

**Affiliations:** 1 Rheumatology, Institute of Child Health, UK; 2 Rheumatology, Great Ormond Street Hospital, London, UK; 3 Rheumatology, Karolinska University Hospital, Karolinska Institue, Sweden; 4 2Institute for Environmental Medicine, Karolinska Institue, Stockholm, Sweden; 5 Dermatology, Philadelphia VAMC and Hospital of the University of Pennsylvania, Philadelphia, USA; 6 Neurology, Academic Medical Centre, Amsterdam, Netherlands; 7 Institute for Environmental Medicine, Karolinska Institue, Stockholm, Sweden; 8 Neurology, Brigham and Women’s Hospital, Boston, USA; 9 Neurology, University of Kansas Medical Center, Kansas City, USA; 10 Rheumatology, Immunology and Allergy, Brigham and Women’s Hospital, Boston, USA; 11 University of Alabama and VA Medical Center, Birmingham, AL, USA; 12 Environmental Autoimmunity Group, Program of Clinical Research, National Institute of Environmental Health Sciences,, National Institutes of Health, US Department of Health and Human Services, Bethesda, USA

## Introduction

Classification criteria are needed to aid recruitment of appropriate patients into research studies. The International Myositis Classification Criteria Project (IMCCP) was set up with support from ACR and EULAR.

## Objectives

To develop and validate new classification criteria for adult and juvenile IIM.

## Methods

Candidate criteria variables were taken from published criteria and inclusion criteria from clinical trials of myositis. Comparator groups confused with IIM were defined. Clinical and laboratory data from IIM and comparator patients were collected from 47 rheumatology, dermatology, neurology and pediatrics clinics worldwide from 2008-2011.

Pair-wise associations among all items and between each item and clinicians’ diagnoses were assessed. Three approaches for derivation of classification criteria were explored: Traditional, Probability score and Classification tree.

Internal validation using bootstrap methods and external validation using data from the Euromyositis register and the Juvenile Dermatomyositis cohort biomarker study and repository UK and Ireland was performed.

## Results

976 IIM (74% adults; 26% children) and 624 comparators (81% adults; 19% children) were obtained.

The new criteria comprise clinical items on muscles, skin, and laboratory measures. Muscle biopsy features can be included. Each item has an assigned score, the total score corresponds to the probability of having IIM. Each probability has specific sensitivity/specificity measures making it possible to use individual inclusion criteria for clinical studies. If no skin rash is present a muscle biopsy is mandatory. High probability of IIM is considered if the score > 7.5 (or > 8.7 if no skin rash), with minimum probability cutoff of 50% (score 5.3 or 6.5). Table 1.

**Table 1:** Performance of new and exisiting classification / diagnostic criteria for idiopathic inflammatory myopathies

Performance (%)	New classification criteria^a^		Peter & Bohan [1]^b^	Tanimoto *et al.* [2]	Targoff *et al.* [3]^b^	Dalakas & Hohlfeld [4]^b^	Hoogendijk *et al.* [5]^b^
						
	Without muscle biopsy data	With muscle biopsy data					
Sensitivity	91	94	98	96	93	6	51
Specificity	82	85	55	31	88	99	96
Correctly classified	88	91	86	79	91	45	70

^a^ Cut point for probability: 55%

^b^ Definite and probable polymyositis and dermatomyositis

External validation using data on 592 adult or 332 juvenile IIM patients yielded 100% sensitivity.

## Conclusion

The new classification criteria for IIM have easy-to-access items and show generally superior performance compared to existing criteria. Approval for these will be sought from ACR/EULAR.

## Disclosure of interest

None declared.

